# The Utility of Sperm DNA Fragmentation as a Diagnostic Tool for Male Infertility and Its Predictive Value for Assisted Reproductive Technology Outcomes

**DOI:** 10.3390/ijms26136314

**Published:** 2025-06-30

**Authors:** Coral Zurera-Egea, Sílvia Mateo, Sergi Novo, Marta Asensio, Montserrat Boada, Marta Antich, Sergi Rovira, Zaida Sarrate, Joan Blanco, Ester Anton

**Affiliations:** 1 Genetics of Male Fertility Group, Unitat de Biologia Cel lular, Departament de Biologia Cel lular, de Fisiologia i d’Immunologia, Universitat Autònoma de Barcelona, 08193 Cerdanyola del Vallés, Spain; coral.zurera@uab.cat (C.Z.-E.); zaida.sarrate@uab.cat (Z.S.); 2 Dexeus Fertility, Department of Obstetrics, Gynecology and Reproductive Medicine, Hospital Universitari Dexeus, 08028 Barcelona, Spain; 3 Fertilab, 08017 Barcelona, Spain; sergi.novo@fertilab.org (S.N.);; 4 Reproclinic, 08011 Barcelona, Spain

**Keywords:** SDF, infertility, seminal alterations, fertilization, blastocyst formation, embryo quality, implantation

## Abstract

Standard semen parameters remain the cornerstone of male infertility evaluation, though they often poorly reflect the likelihood of success in assisted reproductive technology (ART). This study evaluates sperm DNA fragmentation (SDF) as a diagnostic tool for male infertility and predictive biomarker for ART success. Semen samples were collected from 20 fertile donors and 40 infertile patients with abnormal semen parameters. A fraction of each sample was used for SDF assessment via TUNEL assay and flow cytometry, while the remaining portion was processed for conventional semen analysis and ART. Infertile patients exhibited higher SDF levels (32.77 ± 13.61%) compared to donors (22.19 ± 8.37%; *p* < 0.01), a difference that remained statistically significant across all subgroups stratified by semen parameters. Additionally, significant correlations were obtained between the percentage of SDF and sperm count (*r* = −0.4036), motility (*r* = −0.6377), and morphology (*r* = −0.2783). Regarding ART outcomes, patients with low-quality embryos exhibited higher SDF levels compared to those with high-quality embryos (30.02 ± 12.52% vs. 23.16 ± 8.41%; *p* = 0.0036). Receiver operating characteristic (ROC) curve analysis revealed an area under the curve (AUC) above 0.7 for the classification of male infertility as well as the assessment of embryo quality. Overall, our results support the utility of SDF as both a diagnostic biomarker for male infertility and a predictive indicator of embryo quality in ART, particularly in the presence of an oocyte-related female factor.

## 1. Introduction

Infertility is an uprising social and economic issue in modern society, with an estimated 15% worldwide prevalence among individuals of reproductive age [1,2,3]. Assisted reproductive technologies (ART) are the gold standard treatment offered to patients with this condition. However, despite its widespread use, only 30–40% of those undergoing ART cycles are likely to achieve a live birth [2]. Male infertility is present, either as a sole cause or as a combined factor, in approximately 50% of infertile couples seeking ART treatment [3,4]. Its management formerly relied on seminal parameter assessment, but this has proven to be insufficient for establishing the cause of infertility. Moreover, seminal parameters often lack correlation with ART-related outcomes [5]. Therefore, the search for new molecular-based determinations to complement the information given in the semen analysis has emerged as a key area of focus. Candidate molecular determinations encompass genomic, transcriptomic, and epigenomic studies, among others [6,7].

Among these factors, sperm DNA fragmentation (SDF) stands out, as multiple studies suggest it is effective in distinguishing infertile patients from fertile donors, who typically exhibit lower SDF values [8,9,10,11,12,13,14]. SDF has also been reported in numerous studies to correlate with several ART-related outcomes such as fertilization rate, embryo quality [15,16], pregnancy rate, and spontaneous abortion rates [12,17,18,19,20,21], as well as a potential association with live birth rate [21]. In contrast, other studies deny some of these correlations, leading to inconsistencies regarding the potential predictive value of SDF for ART performance [15,22]. This controversy and inconsistency in reports may be caused by the divergence in cut-off values established to determine patients with positive results. While some studies use an arbitrary cut-off of 30% or even higher [16,18,19,20], others set this value lower than 20% [15,21]. Setting a correct cut-off value may allow an accurate diagnostic inference, identifying SDF as a potential cause of infertility and improving the prediction of ART success [8,9,18,23,24,25,26,27,28]. Accordingly, the last World Health Organization (WHO) manual advised the use of SDF assessment in the clinical setting, considering that each fertility center must establish their own threshold values based on their control population [29].

Additionally, the technique used for SDF assessment may represent another significant source of variability that affects the integration of all published results. This stems from the wide range of techniques used in the literature due to the lack of a gold standard. The most used techniques are sperm chromatin structure assay (SCSA), sperm chromatin dispersion (SCD), comet assay, and terminal deoxynucleotidyl transferase dUTP nick-end labeling (TUNEL) assay [30]. Each technique has different standardization levels and equipment requirements and allows the detection of SDF through different approaches. Hence, each laboratory selects the techniques that are best suited to their specific conditions and resources [30].

In this study, we expand on the role of SDF as both a diagnostic and prognostic tool in ART. To this end, we evaluated SDF through TUNEL assay using flow cytometry in spermatozoa from a cohort of fertile semen sample donors (controls, *n* = 20) and a cohort of infertile patients with abnormal semen parameters (*n* = 40). For its diagnostic performance, results were analyzed in conjunction with seminal parameters, including sperm count, motility, and morphology. Additionally, SDF results were also compared to ART-related outcomes such as fertilization, blastocyst formation, embryo quality, and implantation rates. Therefore, the main goals of this study were to test the diagnostic value of SDF and its relationship to seminal parameters and to elucidate its predictive potential regarding different ART-related outcomes.

## 2. Results

The analysis of SDF in controls showed a mean ± standard deviation (SD) of 22.19 ± 8.37% with a 95% confidence interval (CI) ranging from 18.27% to 26.11% (Supplementary Table S1). Using the upper limit of this interval, we established the SDF threshold for our study. Setting an SDF cut-off value of 26% allowed the classification of the study participants in high SDF (>26%) or low SDF (≤26%) groups. Among the controls, 70% (14/20) were classified in the low SDF group (see Supplementary Table S1 for a detailed view of SDF results in fertile donors, along with their corresponding threshold-based classification as high/low).

The analysis of SDF in infertile patients showed a mean ± SD of 32.77 ± 13.61%. Out of all patients in the study, 60% (24/40) were classified in the high SDF group (See Supplementary Table S2 for a detailed view of SDF results in infertile patients, along with their corresponding threshold-based classification as high/low).

### 2.1. SDF, Seminal Parameters, and Age: Correlation Results

**Figure 1:**
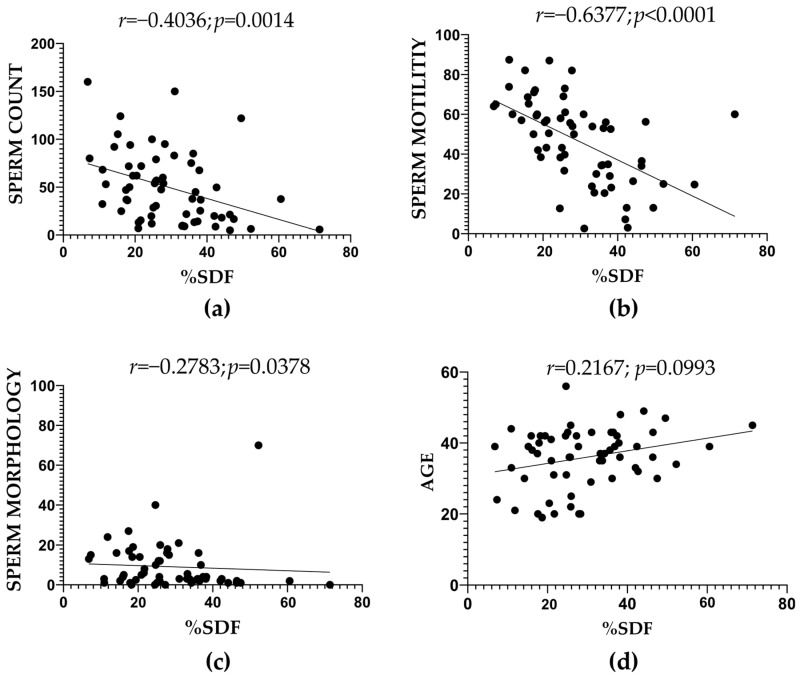
Results of the correlation between SDF content and (**a**) sperm count (Million/mL), (**b**) sperm motility (% of progressive motility), (**c**) sperm morphology (% of normal forms), and (**d**) age (years).

Correlations between SDF levels and sperm parameters were analyzed, including data from both the control group (Supplementary Table S1 and Table 1) and infertile patients (Supplementary Table S2 and Table 2). Significant negative correlations were observed with sperm count (*r* = −0.4036; *p* = 0.0014), sperm motility (*r* = −0.6377; *p* < 0.0001), and sperm morphology (*r* = −0.2783; *p* = 0.0378) (Figure 1a, Figure 1b, and Figure 1c, respectively). In contrast, no significant correlation was observed between SDF content and age (*r* = 0.2167; *p* = 0.0993) (Figure 1d).

### 2.2. SDF, Seminal Parameters, and Age: Results of Group Comparisons

**Figure 2:**
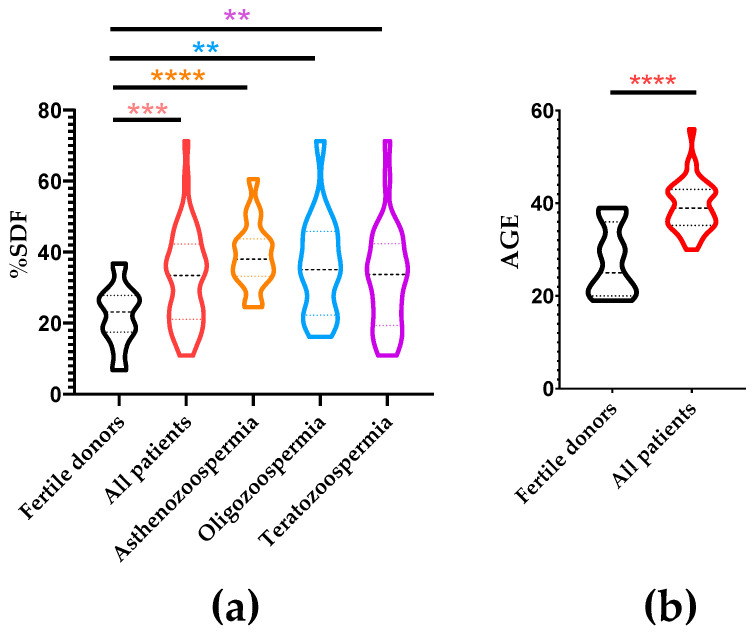
SDF levels among participant cohorts. (**a**) Comparison of SDF levels between fertile donors and patient subgroups according to their seminal parameters. (**b**) Comparison of age between fertile donors and patients. ** Indicate *p*-values under 0.01. *** Indicate *p*-values under 0.001. **** Indicate *p*-values under 0.0001.

The analysis of SDF revealed a significant increase in the mean ± SD in the infertile population (32.77 ± 13.61%) compared to the control group (22.19 ± 8.37%; *p* < 0.001). When considering data from stratified groups of infertile patients, all of them exhibited significantly higher mean ± SD of SDF compared to the control group: oligozoospermic (36.37 ± 14.30%; *p* = 0.0019), asthenozoospermic (39.27 ± 9.50%; *p* < 0.0001), and teratozoospermic (32.95 ± 14.66%; *p* = 0.0028) (Figure 2a).

The evaluation of differences in mean ± SD age between fertile donors and infertile patients revealed a significantly increased age in the infertile population (27.58 ± 7.43 years) compared to fertile donors (39.43 ± 5.34 years; *p* < 0.0001) (Figure 2b).

### 2.3. SDF and Seminal Parameters: Diagnostic Value Results

**Figure 3:**
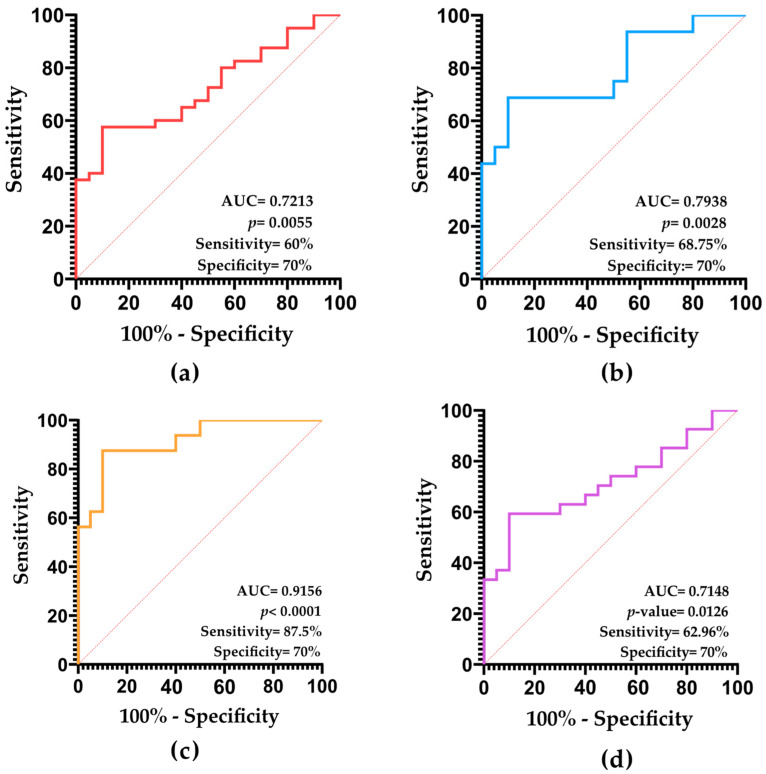
Receiver operating characteristic (ROC) curve indicating the potential of SDF as a diagnostic biomarker for male infertility for (**a**) all individuals, (**b**) oligozoospermia, (**c**) asthenozoospermia, and (**d**) teratozoospermia.

We evaluated the potential of SDF as a diagnostic biomarker using receiver operating characteristic (ROC) curves. When applied to all individuals included in the study, the diagnostic accuracy, as evaluated by the area under the curve (AUC), was 0.7213 (*p* = 0.0055), indicating a fair performance (Figure 3a). Using the cut-off value of 26% (Supplementary Table S1), we obtained a 60% sensitivity, a 70% specificity, an 80% positive predictive value (PPV), and a 46.67% negative predictive value (NPV) for the diagnosis of male infertility based on SDF levels (Figure 3a).

In the evaluation of SDF as a diagnostic biomarker for specific groups of infertile patients with seminal alterations, ROC curve analyses revealed the following: AUC = 0.7938 for oligozoospermia (*p*-value < 0.01; fair diagnostic accuracy) (Figure 3b), AUC = 0.9156 for asthenozoospermia (*p*-value < 0.0001; excellent diagnostic accuracy) (Figure 3c), and AUC = 0.7148 for teratozoospermia (*p*-value = 0.0126; fair diagnostic accuracy) (Figure 3d).

### 2.4. SDF and ART-Related Outcomes: Correlation Results

**Figure 4:**
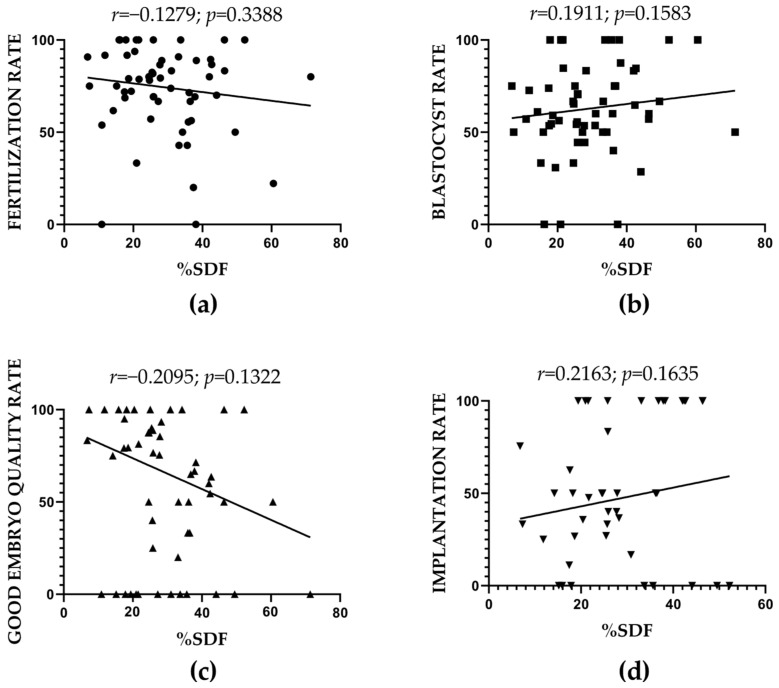
Correlation between SDF levels and assisted reproductive technology (ART)-related outcomes. (**a**) Fertilization rate, (**b**) blastocyst rate, (**c**) good embryo quality rate, and (**d**) implantation rate.

Data from both control and patient groups were considered in the correlation analysis between percentage of SDF (Supplementary Tables S1 and S2) and ART outcomes, including fertilization, blastocyst formation, embryo quality, and implantation rates (Table 1 and Table 2). No significant associations were observed with any of the parameters evaluated (Figure 4).

### 2.5. SDF and ART-Related Outcomes: Results of Group Comparisons

**Figure 5:**
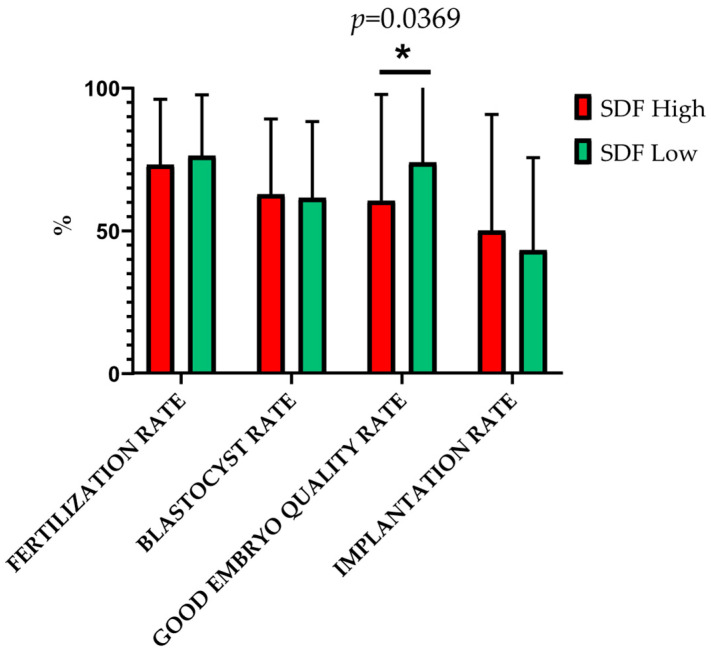
Mean ± SD differences in ART-related outcomes according to SDF group classification. * Indicate *p*-values under 0.05.

The analysis of ART-related outcomes based on participant classification according to SDF content revealed a significantly lower mean ± SD of good embryo quality rate in participants with high SDF (60.31 ± 37.27%) compared to those with low SDF (74.19 ± 35.75%; *p* = 0.0369). Conversely, we observed no significant differences in mean ± SD values between these groups regarding fertilization rate (*p* = 0.5058), blastocyst rate (*p* = 0.8519), and implantation rate (*p* = 0.4810) (Figure 5).

**Figure 6:**
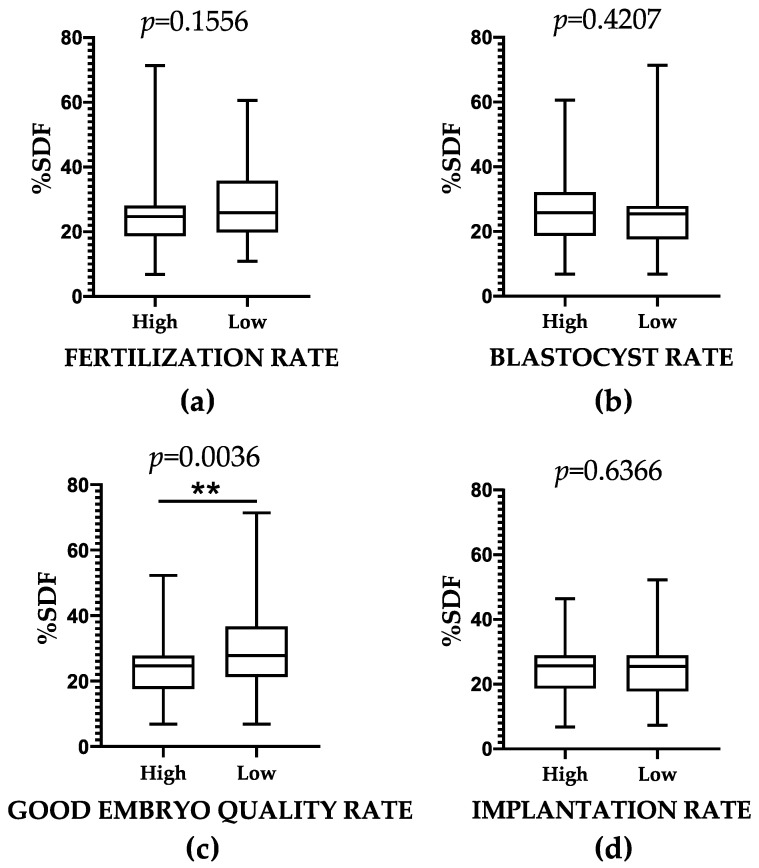
Differences in the mean ± SD percentage of SDF according to (**a**) fertilization rate, (**b**) blastocyst rate, (**c**) good embryo quality rate, and (**d**) implantation rate. ** Indicate *p*-values under 0.01.

To further investigate the relationship between SDF and ART outcomes, we compared the mean ± SD of SDF across groups categorized by their ART performance. To facilitate this, ART-outcome thresholds of normality were established by calculating the lower limit of the 95% CI of the mean for each ART-related outcome, based on data from ART cycles involving both donor sperm and donor oocytes (Table 1; See Column: Female Fertility Status; Category: Fertile donor). These threshold values, representing the outcomes in couples without infertility, were 69% for fertilization rate, 51% for blastocyst rate, 75% for good embryo quality rate, and 37% for implantation rate (Table 1). The comparison of mean ± SD of SDF between groups based on each ART-related outcome performance (high/low) revealed no significant differences for fertilization rate, blastocyst rate, and implantation rate (*p* > 0.05) (Figure 6). Nonetheless, we observed a significantly increased mean ± SD of SDF in the low embryo quality rate group compared to the high embryo quality rate group (30.02 ± 12.52% vs. 23.16 ± 8.41%; *p* = 0.0036).

### 2.6. SDF and ART-Related Outcomes: Predictive Value Results

**Figure 7:**
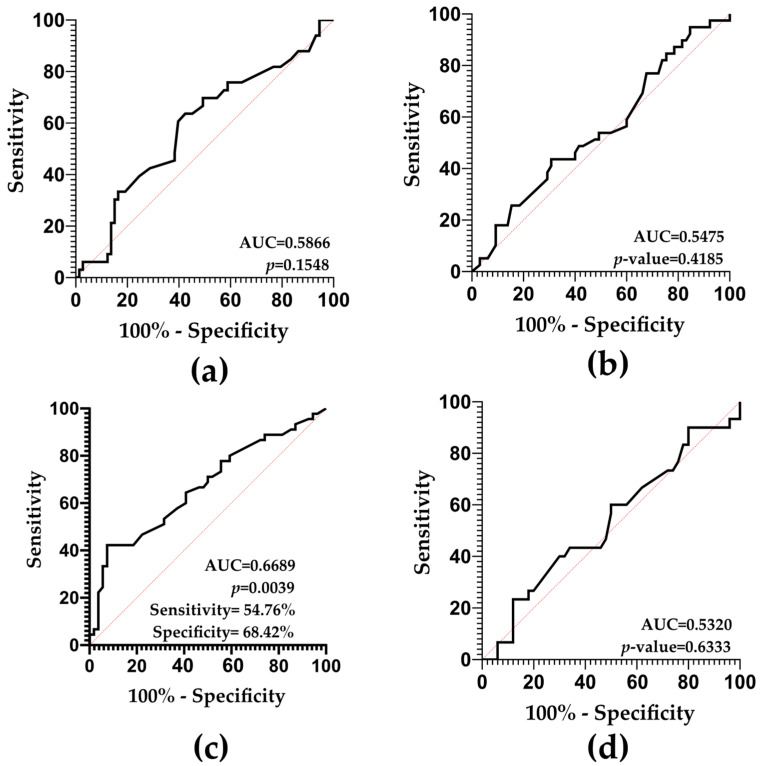
ROC curves indicating the potential of SDF as a predictive biomarker of (**a**) fertilization rate, (**b**) blastocyst rate, (**c**) good embryo quality rate, and (**d**) implantation rate.

ROC curve analyses based on ART performance did not reveal any predictive potential of SDF for fertilization rate (AUC = 0.5866; *p*-value = 0.1548), blastocyst rate (AUC = 0.5475; *p*-value = 0.4185), or implantation rate (AUC = 0.5320; *p*-value = 0.6333) (Figure 7a,b,d).

Conversely, SDF was found to be a potential predictor of good embryo quality rate (AUC = 0.6689; *p*-value = 0.0039), as depicted in Figure 7c. This analysis yielded a 54.76% sensitivity, a 68.42% specificity, a 51.11% PPV, and a 61.90% NPV.

## 3. Discussion

### 3.1. SDF and Seminal Parameters: Diagnostic Value

Multiple previous reports indicate that SDF content measured through TUNEL assay is significantly increased in infertile patients compared to fertile males [8,9,11,13,23,31,32,33]. Our study, conducted with a total cohort of 60 individuals, further supports the observation of an elevated SDF percentage in infertile individuals. This finding was further validated through ROC analysis, which yielded an AUC greater than 0.7, indicating that SDF is a reliable biomarker for the diagnosis of male infertility.

In this context, most studies agree on the diagnostic potential of SDF assessment using the TUNEL assay; however, AUC values may vary slightly between studies, likely reflecting methodological differences or variations in study populations. For instance, in the studies conducted by Sharma R. et al. [8], Sergerie M. et al. [9], Ribas-Maynou J. et al. [31], and Kabartan E. et al. [32] AUC values over 0.8 were obtained, supporting the use of TUNEL for assessing SDF as a biomarker with good to excellent diagnostic potential. However, the studies by Muratori et al. [13] and Voncina SM. et al. [33] observed lower AUCs (0.757 and 0.7, respectively), indicating a fair diagnostic potential, while Punjabi U. et al. [11] and Sharma R. et al. [23] observed AUCs indicating a poor or failed diagnostic potential (0.608 and 0.556, respectively).

Several factors may account for the variability observed. One key variable is cohort size, which significantly influences the strength of the results. Larger sample sizes enhance statistical power and reduce the likelihood of bias, making the results more robust and applicable to broader clinical populations. Many studies reporting higher diagnostic accuracy for the TUNEL assay involved cohorts of over 100 infertile patients [8,11,13,23,31], whereas our study was based on a comparatively smaller sample of 60 individuals. This disparity in sample size may partly explain differences in AUC values and overall diagnostic conclusions.

Another critical factor is cohort selection criteria, particularly the inclusion of patients based on specific infertility phenotypes. The heterogeneity of infertility etiologies can influence SDF levels and the assay’s diagnostic sensitivity. Studies that focus on more narrowly defined patient groups may yield different outcomes compared to those that include a broader or more heterogeneous infertile population. In this sense, when in our study infertile patients were stratified by specific semen parameter abnormalities, all subgroups exhibited higher SDF percentages compared to fertile individuals. These differences were further proven when performing ROC analyses, highlighting the importance of phenotype-specific evaluation in the clinical interpretation of SDF measurements.

These findings align with previous reports, which also identified sperm motility as the seminal parameter most consistently associated with elevated SDF content [20,25,34,35,36,37,38]. The increased SDF level in asthenozoospermic patients has been associated with an elevated ROS production [35,38,39], a well-documented contributor to sperm DNA damage, which can lead to impaired cellular ATP production—an essential factor for achieving optimal motility [40,41].

Regarding oligozoospermia, the negative correlation between SDF and sperm count has been proposed to be linked to underlying cellular processes, such as abortive apoptosis or nuclease activity. Abortive apoptosis is a specific mechanism of sperm cell death suggested to induce SDF during early stages of sperm differentiation due to poor sperm maturation and inadequate chromatin compaction [42,43,44,45]. Moreover, poor chromatin compaction can be a result of defective sperm maturation due to nuclease activation without subsequent proper re-ligation, leading to the observed increased DNA fragmentation [45,46]. At the same time, poorly compacted DNA is more accessible for nucleases, which can further contribute to DNA fragmentation [47,48]. Together, these mechanisms suggest that patients with sperm maturation defects are more likely to exhibit low sperm counts with high levels of SDF.

In contrast, the association between SDF and sperm morphology remains less straightforward. While our study found a correlation, conventional assessments based on the percentage of normal forms often fail to show a consistent association [25,34]. However, recent evidence supports a more complex interplay between these factors. For instance, studies by Verón L. et al. [25] and Yoshikawa-Terada K. et al. [34] examined specific morphological characteristics such as head width, flagellum length, and midpiece abnormalities and identified significant correlations between these detailed structural parameters and SDF levels. In contrast, neither study reported an association between SDF and the percentage of morphologically normal forms using standard criteria. In our cohort, although a correlation between sperm morphology and SDF was observed, it was notably weaker than the associations seen with sperm count and motility. These findings support the notion that a more detailed, non-conventional morphological assessment may uncover stronger and more clinically meaningful correlations with SDF.

Another factor to consider is the age of participants. In our study, no significant correlation was found between age and SDF content. However, the correlation coefficient and *p*-value suggest a possible trend toward a positive association. This aligns with previous studies that have reported significant correlations between age and SDF values [24,25,34,36,49,50,51]. While some hypothesized this correlation to be a consequence of a poor healthy sperm selection in the testes linked to a failure in triggering apoptosis with aging [50], others believe it may be a consequence of the reported increase in oxidative stress with age [51].

### 3.2. SDF and ART: Predictive Value

The predictive potential of SDF has been evaluated in a few studies by assessing the relationship of SDF with ART-related parameters. However, the results reported in the literature are not entirely consistent [52,53,54]. Again, differences in sample size can have a substantial impact on the results obtained. In our study, the number of analyzed samples was relatively limited, which may affect the statistical power of the findings and could partly account for discrepancies with previously published results. Moreover, a notable additional factor influencing the variability in these findings is the inclusion of couples with female factor infertility, as in our study. This inclusion limits the ability to draw a direct and conclusive relationship between ART outcomes and paternal SDF. Consequently, the potential influence of female infertility factors must be considered when interpreting the results of studies examining SDF’s predictive value.

In our study, we comprehensively evaluated the predictive value of SDF for ART outcomes by systematically assessing its association with several key clinical parameters. Firstly, with respect to the fertilization rate, there are some studies suggesting its relationship with SDF [55,56], while others contradict these findings [57,58,59]. Studies against such correlation, including our own, argue that the paternal genome is not activated until day 3 after fertilization (between the 4-cell and the 8-cell stage). Therefore, sperm DNA integrity cannot hinder the formation of the zygote pronuclei [18,60,61,62].

Concerning blastocyst formation, there are fewer studies evaluating the relationship between the percentages of SDF and blastulation rates, and results reported are partially contradictory [18,56,61]. For instance, Simon L. et al. (2014) [56] observed a correlation between the percentage of blastocysts and SDF levels measured through Comet assay, whereas when using TUNEL, this correlation was not significant. Similarly, our results did not show a significant association between SDF levels assessed by TUNEL and blastocyst formation rates. On the other hand, Zheng W. et al. [61] reported a correlation between blastulation and SDF measured through SCD. Blastocyst formation can also be assessed qualitatively through the analysis of embryo quality. In this regard, most studies agree that there is a correlation between embryo quality rate and SDF values [55,59,61]. These studies suggest that the activation of paternal DNA at day 3 after fertilization, when highly fragmented, disturbs the quality of the resulting embryos [18,60,61,62]. This would explain the decreased good embryo quality rate observed in high SDF participants compared to the low SDF cohort. Taken together, our results suggest that, although SDF seems to not affect the process of blastulation quantitatively, it does impact embryo development in terms of quality. Moreover, our ROC curve analyses revealed a statistically significant, although poor, predictive potential of SDF regarding the good embryo quality rate parameter when analyzing the whole cohort of participants.

To better understand this relationship, we conducted an additional ROC analysis focusing exclusively on ART cycles in which a female factor attributed to oocyte quality was identified (Table 1 and Table 2; Column Oocyte Factor). Under these conditions, the AUC increased to values exceeding 0.7, suggesting improved predictive accuracy. Conversely, when analyzing data from cycles in which no oocyte-related female factor was present, the AUC values decreased even further compared to those obtained from the entire cohort. These observations are in line with findings from Esbert M. et al. [59], who also reported no significant association between SDF content and embryo quality in the absence of oocyte-related factors. A plausible explanation for these results lies in the oocyte’s intrinsic ability to repair DNA damage following fertilization [19]. Therefore, the increased predictive value observed in the presence of an oocyte-related female factor may be attributed to a reduced capacity of these oocytes to repair SDF, thereby allowing the detrimental effects of elevated SDF levels to impact embryo development.

Regarding implantation, similar to other ART-related parameters discussed earlier, there is considerable controversy surrounding the results obtained. Several studies support the impact of SDF on these processes by reporting significantly higher SDF in couples not achieving clinical pregnancy [18,57,58,63,64] and significant correlations between such ART-related outcomes and SDF values [61]. Some studies have also corroborated these results through the direct observation of predictive values through ROC analyses. Nonetheless, other studies deny the predictive value of SDF on implantation when evaluating either specific cohorts based on female factor status or non-selected patient cohorts [59,65,66,67]. Such studies report very low and not statistically significant AUC values, similarly to those observed in the present manuscript. Clearly, the limited sample size may have an influence on our results, since many of the infertile couples (*n* = 15) did not achieve enough good-quality embryos for transfer, and consequently, no data on implantation was available.

Finally, it is worth noting that beyond the key ART parameters discussed above, several studies have reported correlations between SDF values and additional ART outcomes such as spontaneous abortion rates [55,59], as well as live birth rates [21,57,65]. However, in the present study, the number of pregnancies achieved was insufficient to allow for a meaningful evaluation of either outcome, limiting our ability to assess the predictive value of SDF for these endpoints.

## 4. Materials and Methods

### 4.1. Study Cohort Selection

The control group included 20 semen bank donors from the Fertilab center. Control samples were used in several ART cycles either for a single or multiple female recipients. Controls were aged between 18 and 39 years old, all with a normal karyotype, normal semen parameters, and proven fertility (Table 1).

The population of interest consisted of 40 males from infertile couples seeking ART treatment at the DexeusDona (*n* = 35) and Reproclinic Barcelona (*n* = 5) centers. Patients’ ages range between 30 and 56 years old, and all of them presented normal karyotypes. Inclusion criteria required two consecutively altered semen analysis results (WHO guidelines [29]), irrespective of the female partner’s condition (Table 2).

The study was approved in accordance with the Declaration of Helsinki by the Research Ethics Committee of the Universitat Autònoma de Barcelona (No. 5921) on 20 May 2022, as well as by the internal ethics committees of the collaborating centers. All participants provided written informed consent for secondary use of their semen sample and ART-related data.

### 4.2. Semen Sample and Data Collection

Semen samples were collected by masturbation after 3–7 days of sexual abstinence. A fraction of each sample was cryopreserved with sperm freezing medium (CooperSurgical, Trumbull, CT, USA) in a 1:1 proportion until its use for SDF determinations (control and infertile samples) or ART treatments (control samples only). The remaining portion of the same samples was used for seminal parameter analyses (both control and infertile samples) and to perform the corresponding ART cycles (infertile samples only). Regarding the evaluation of seminal parameters, we collected data about sperm count, sperm morphology, and progressive sperm motility. For ART-related parameters, collected data included fertilization rate, defined as the proportion of zygotes relative to the total number of inseminated oocytes; blastocyst rate, defined as the proportion of blastocysts formed relative to the total number of zygotes; good embryo quality rate, defined as the proportion of grade A and grade B blastocysts—that is, blastocysts with the highest potential for implantation and successful pregnancy according to ASEBIR criteria [68]—relative to the total number of blastocysts obtained; and implantation rate, defined as the number of intrauterine gestational sacs regarding the total number of embryo transfers performed (Table 1 and Table 2). It is important to note that oocytes used in ART cycles for both patient and control semen samples were derived from women with diverse fertility status, including both fertile and infertile women.

### 4.3. Sperm DNA Fragmentation Measurement Procedure

SDF assessment was performed through a TUNEL assay, using the In Situ Cell Death Detection Kit (Roche Diagnostic GmbH, Penzberg, Germany). This kit enables the enzymatic incorporation of fluorescein-labeled dUTP nucleotides into free 3′-OH ends. Samples were thawed and processed for TUNEL according to the manufacturer’s instructions. Briefly, sperm were washed in phosphate-buffered saline (PBS), permeabilized with triton X-100 and fixed in paraformaldehyde. After fixation, the TUNEL reaction was performed under three different conditions to ensure proper assay validation and interpretation. A negative control was used, consisting of spermatozoa not exposed to labeled dUTP nucleotides. A positive control was used, which involved spermatozoa exposed to DNase I to induce DNA fragmentation (1000 U/mL; Roche Diagnostic GmbH, Penzberg, Germany). The test sample included spermatozoa exposed to the TUNEL reaction with labeled dUTP.

### 4.4. Flow Cytometry Acquisition and Analysis

Samples were analyzed through flow cytometry with CytoflexS (Beckman Coulter, Inc. Brea, California, USA) using the CytExpert (Version: 2.5.0.77) software, which allowed us to distinguish SDF+ from SDF− cells according to the pattern of labeling (Supplementary Figure S1).

Acquired events were first plotted based on forward scatter height (FSC-H) and side scatter (SSC) to identify and gate sperm-like cells (Supplementary Figure S1a). Next, these gated events were plotted for FSC-H and forward scatter area (FSC-A) to exclude cell aggregates (Supplementary Figure S1b). Finally, an additional plot of SSC versus phycoerythrin (PE) detector channel (585/42 BP; Propidium iodide staining) was performed to select individual sperm cells while excluding apoptotic bodies and debris (Supplementary Figure S1c) [69].

To differentiate positive from negative cells, sperm cells were plotted using a histogram for the Fluorescein Isothiocyanate (FITC) detector channel (585/40 BP; fluorescein-labeled dUTP staining), which labeled DNA fragmentation. To establish sample-specific thresholds, both the positive (Supplementary Figure S1f) and negative controls (Supplementary Figure S1e) were analyzed alongside the test sample (Supplementary Figure S1d). This approach enabled us to determine the threshold for labeling intensity and segregate SDF+ and SDF− cells to obtain a percentage of SDF.

### 4.5. Statistical Analysis

Statistical analyses were performed through GraphPad Prism version 8.0.1 (GraphPad Software, Boston, MA, USA). Different approaches were taken depending on the objective aimed to achieve and data distribution (parametric or non-parametric):

#### 4.5.1. SDF, Seminal Parameters, and Age: Correlations Assessment

To establish the correlation between the percentage of SDF and sperm parameters, as well as between SDF levels and age, Pearson’s R or Spearman’s Rho were performed depending on the data distribution. These analyses were conducted for the entire cohort of individuals, including donors and patients.

#### 4.5.2. SDF, Seminal Parameters, and Age: Group Differences Assessment

The mean ± SD differences in the percentage of SDF between fertile individuals and infertile patients were assessed using either Student *t*-tests or Mann–Whitney U-tests, depending on the data distribution. Fertile donors were compared to both the entire infertile population and specific subgroups of infertile males classified by seminal parameters. In cases where patients exhibited multiple seminal alterations, they were assigned to several groups accordingly. Welch’s correction was applied to those Student *t*-test comparisons in which groups had significantly different variances. Additionally, age mean ± SD differences between fertile individuals and infertile patients were assessed using Mann–Whitney U-tests.

#### 4.5.3. SDF and Seminal Parameters: Predictive Value Assessment

To assess the potential of SDF as a diagnostic biomarker of male infertility, ROC curves analyses were performed. AUC values were interpreted as follows: Excellent (0.9 ≤ AUC ≤ 1), Good (0.8 ≤ AUC < 0.9), Fair (0.7 ≤ AUC < 0.8), Poor (0.6 ≤ AUC < 0.7) and Failed (AUC < 0.6) [70]. The cut-off value for discriminating high/low SDF groups of individuals was established as the truncated value of the upper limit of the 95% CI for the mean ± SD of SDF in fertile donors (see data in Supplementary Table S1). Sensitivity (True Positive Rate), specificity (True Negative Rate), PPV and NPV, were evaluated.

#### 4.5.4. SDF and ART-Related Outcomes: Correlations Assessment

To evaluate the correlation between SDF values and ART-related outcomes (fertilization, blastocyst formation, good embryo quality rate, and implantation rate), Pearson’s R or Spearman’s Rho were performed depending on the data distribution. When multiple ART cycles were performed using oocytes from different women, as in the case of control samples (see data in Table 1), multiple data points per male donor were included in the analysis.

#### 4.5.5. SDF and ART-Related Outcomes: Group Differences Assessment

To test the influence of SDF on ART performance, quantitative outcome values were transformed into two categorical variables using specific cut-off values (see data in Supplementary Table S1 and Table 1). Threshold values were determined based on the truncated lower limit of the 95% CI of the mean for each ART-related outcome. This was derived from ART cycles using donor sperm and donor oocytes, as this group was considered to have the optimal ART performance (see data on Table 1). Data between groups were compared using Student *t*-tests or Mann–Whitney U-tests, depending on the data distribution.

#### 4.5.6. SDF and ART-Related Outcomes: Predictive Value Assessment

To evaluate the potential of SDF as a predictive biomarker for ART-related outcomes, ROC curve analyses were performed and interpreted according to the criteria outlined in Section 4.5.3. When statistical significance was established, positive predictive value (PPV), negative predictive value (NPV), sensitivity, and specificity were subsequently assessed.

## 5. Conclusions

This study reinforces the diagnostic value of sperm DNA fragmentation, particularly when assessed via TUNEL assay, as a biomarker of male infertility. Our results align with previous literature showing elevated SDF in infertile men with seminal alterations and support its clinical utility, with ROC analysis yielding an AUC above 0.7. In the context of assisted reproductive technologies (ART), our findings suggest that while SDF may not significantly impact fertilization, blastocyst formation, and implantation rates, it compromises embryo quality. Importantly, the predictive value of SDF improves in the presence of female oocyte-related factors, likely due to reduced oocyte DNA repair capacity.

## Figures and Tables

**Table 1:** Seminal parameters and ART-related outcomes for each fertile donor included in the study. The mean value, the 95% confidence interval (CI) limits, and the cut-off value derived for binary classification (high/low) of each ART-related outcome are specified. These figures were calculated based on data from cycles in which oocytes from fertile donors were used.

Control Subject ID	Sperm Count (Mill/mL)	Sperm Motility (% Progressive Motility)	Sperm Morphology (% Normal Forms)	Female Fertility Status	Oocyte Factor	Fertilization Rate (%)	Blastocyst Rate (%)	Good Embryo Quality Rate (% Grade A + B Blastocysts)	Implantation Rate (%)
D1 ^#^	72.00	87.00	8.00	Fertile donor	NO	57.14	100.00	75.00	42.86
				Infertile (AMA)	YES	88.89	87.50	85.71	50.00
				Infertile (AMA)	YES	90.00	66.70	83.33	50.00
D2 ^#^	100.00	58.00	10.00	Fertile donor	NO	81.25	30.80	75.00	66.67
				Fertile	NO	75.00	100.00	100.00	33.33
D3	53.00	60.00	24.00	Fertile donor	NO	91.67	72.70	100.00	25.00
D4 ^#^	47.00	50.00	27.00	Fertile donor	NO	72.22	61.50	37.50	33.33
				Infertile (AMA)	YES	60.00	100.00	100.00	0.00
				Infertile (AMA)	YES	83.33	60.00	100.00	0.00
D5 ^#^	79.00	73.00	12.00	Fertile donor	NO	75.00	66.70	66.67	100.00
				Fertile donor	NO	70.00	85.70	100.00	66.67
				Fertile donor	NO	62.50	60.00	100.00	NA
D6 ^#^	57.00	61.00	20.00	Fertile donor	NO	50.00	100.00	33.33	100.00
				Fertile donor	NO	62.50	20.00	100.00	0.00
				Fertile donor	NO	66.67	75.00	100.00	50.00
				Infertile (AMA)	YES	66.67	100.00	50.00	0.00
				Infertile (AMA)	YES	100.00	57.10	100.00	50.00
D7 ^#^	54.00	69.00	12.00	Fertile	NO	90.48	52.60	60.00	50.00
				Fertile donor	NO	100.00	54.50	100.00	25.00
				Fertile	NO	62.50	60.00	100.00	33.33
				Infertile (AMA)	YES	76.92	50.00	100.00	0.00
D8 ^#^	95.00	50.00	15.00	Fertile donor	NO	66.67	100.00	100.00	40.00
				Infertile (AMA)	YES	100.00	83.30	80.00	33.33
				Infertile (AMA)	YES	100.00	66.70	100.00	NA
D9	50.00	60.00	14.00	NA	NA	NA	NA	NA	NA
D10 ^#^	83.00	60.00	21.00	Infertile (AMA)	YES	77.78	42.90	100.00	50.00
				Infertile (AMA)	YES	63.64	42.90	100.00	0.00
				Infertile (AMA)	YES	80.00	75.00	100.00	0.00
D11 ^#^	45.00	56.00	10.00	Fertile donor	NO	50.00	50.00	50.00	100.00
				Fertile donor	NO	62.50	100.00	80.00	NA
D12	80.00	65.00	15.00	Fertile donor	NO	75.00	50.00	100.00	33.33
D13 ^#^	94.00	42.00	19.00	Fertile donor	NO	100.00	50.00	66.67	0.00
				Fertile donor	NO	71.43	20.00	100.00	0.00
				Infertile (AMA)	YES	85.71	83.30	60.00	0.00
				Fertile	NO	80.00	75.00	100.00	50.00
				Fertile	NO	86.67	61.50	87.50	50.00
				Infertile (AMA)	YES	83.33	100.00	80.00	50.00
				Fertile donor	NO	87.50	71.40	100.00	40.00
				Infertile (AMA)	YES	42.86	0.00	NA	NA
				Infertile (AMA)	YES	81.82	55.60	100.00	50.00
				Infertile (AMA)	YES	100.00	33.30	100.00	0.00
				Infertile (AMA)	YES	50.00	100.00	0.00	NA
D14 ^#^	62.00	56.00	14.00	Fertile donor	NO	87.50	50.00	100.00	71.43
				Fertile donor	NO	100.00	62.50	100.00	0.00
D15 ^#^	54.00	54.00	18.00	Fertile donor	NO	75.00	50.00	66.67	50.00
				Infertile (AMA)	YES	90.00	11.10	100.00	NA
				Infertile (AMA)	YES	66.67	50.00	75.00	NA
				Infertile (AMA)	YES	85.71	66.70	100.00	NA
D16 ^#^	60.00	82.00	16.00	Fertile donor	NO	88.89	62.50	80.00	50.00
				Fertile donor	NO	84.62	45.50	100.00	50.00
				Infertile (AMA)	YES	66.67	50.00	0.00	0.00
				Infertile (AMA)	YES	100.00	33.30	100.00	0.00
				Fertile donor	NO	87.50	100.00	85.71	50.00
				Fertile donor	NO	87.50	57.10	100.00	50.00
				Fertile	NO	76.92	80.00	62.50	80.00
				Fertile donor	NO	100.00	0.00	NA	NA
D17	85.00	53.00	16.00	Fertile	NO	71.43	40.00	50.00	50.00
D18 ^#^	37.00	71.00	17.00	Fertile donor	NO	87.50	42.90	100.00	50.00
				Fertile donor	NO	75.00	50.00	100.00	NA
				Infertile (AMA)	YES	36.84	71.40	80.00	50.00
				Fertile donor	NO	75.00	50.00	100.00	75.00
D19 ^#^	92.00	57.00	16.00	Fertile donor	NO	50.00	40.00	100.00	50.00
				Fertile donor	NO	16.67	100.00	0.00	NA
				Fertile donor	NO	100.00	66.70	100.00	50.00
				Fertile donor	NO	80.00	37.50	100.00	50.00
D20 ^#^	160.00	64.00	13.00	Infertile (AMA)	YES	100.00	100.00	100.00	100.00
				Fertile	NO	92.31	50.00	66.67	66.67
				Fertile donor	NO	80.00	75.00	83.33	60.00
Mean ^##^	NA	NA	NA	NA	NA	75.68	59.94	83.82	47.64
95% CI	NA	NA	NA	NA	NA	69.55–81.80	51.34–68.54	75.16–92.48	37.56–57.72
Cut-off value	NA	NA	NA	NA	NA	69.00%	51.00%	75.00%	37.00%

^#^ Semen samples used for multiple ART cycles involving oocytes from different women, leading to various ART-related outcomes. Each outcome was considered independently and included as a separate data point in all analyses. ^##^ Values calculated using data exclusively from cycles involving oocytes donated by fertile women. NA = not applicable. AMA = advanced maternal age.

**Table 2:** Seminal parameters and ART-related outcomes for each infertile patient included in the study.

Infertile Subject ID	Sperm Count (Mill/mL)	Sperm Motility (% Progressive Motility)	Sperm Morphology (% Normal Forms)	Female Fertility Status	Oocyte Factor	Fertilization Rate (%)	Blastocyst Rate (%)	Good Embryo Quality Rate (% Grade A + B Blastocysts)	Implantation Rate (%)
P1	36.17	72.16	1.00	Infertile (AMA)	YES	100.00	100.00	0.00	0.00
P2	68.20	87.39	1.00	Infertile (Endometriosis)	NO	53.85	57.14	0.00	NA
P3	6.33	25.00	70.00	Infertile (Endometriosis)	NO	100.00	100.00	100.00	0.00
P4	37.78	34.52	3.00	Infertile (AMA)	YES	55.56	60.00	33.33	NA
P5	61.97	38.45	2.50	Infertile (AMA)	YES	72.22	30.77	0.00	100.00
P6	13.17	57.00	NA	Fertile	NO	33.33	100.00	0.00	100.00
P7	75.12	34.31	2.50	Infertile (AMA)	YES	42.86	100.00	0.00	0.00
P8	30.56	39.72	1.90	Fertile	NO	100.00	44.44	25.00	100.00
P9	24.89	65.28	5.00	Infertile (Endometriosis + AMA)	YES	100.00	0.00	NA	NA
P10	36.81	23.19	4.00	Infertile (Endometriosis)	NO	88.89	87.50	71.43	100.00
P11	124.10	68.64	3.92	Infertile (AMA)	YES	100.00	50.00	100.00	0.00
P12	9.47	23.81	3.00	Fertile	NO	90.91	50.00	20.00	100.00
P13	105.31	82.08	2.00	Infertile (Recurrent abortions)	YES	75.00	33.33	0.00	0.00
P14	14.29	34.86	4.00	Infertile (AMA)	YES	20.00	0.00	NA	NA
P15	25.42	52.58	3.00	Infertile (AMA + Low ovarian reserve)	YES	0.00	NA	NA	NA
P16	47.53	55.66	0.00	Infertile (AMA)	YES	66.67	50.00	0.00	NA
P17	13.36	20.41	2.00	Infertile (AMA)	YES	66.67	75.00	33.33	50.00
P18	5.82	60.00	0.00	Infertile (Endometrial polyp + AMA)	YES	80.00	50.00	0.00	NA
P19	9.06	20.60	3.00	Infertile (AMA)	YES	100.00	100.00	0.00	0.00
P20	32.31	73.83	3.00	Infertile (AMA + Low ovarian reserve)	YES	0.00	NA	NA	NA
P21	4.90	36.51	2.00	Infertile (AMA)	YES	83.33	60.00	100.00	100.00
P22	55.68	31.67	4.00	Infertile (AMA)	YES	81.82	55.56	40.00	33.33
P23	16.69	56.20	1.00	Infertile (Low ovarian reserve)	YES	NA	NA	NA	NA
P24	8.79	13.00	3.00	Infertile (AMA)	YES	89.47	64.71	54.55	100.00
P25	21.30	34.04	1.00	Infertile (Ovulatory factor)	YES	100.00	57.14	50.00	100.00
P26	37.52	24.69	1.92	Infertile (AMA)	YES	22.22	100.00	50.00	NA
P27	21.83	30.00	1.00	Infertile (AMA)	YES	50.00	50.00	100.00	NA
P28	67.55	29.00	NA	Infertile (Endometriosis)	NO	69.23	100.00	66.67	100.00
P29	49.70	3.00	NA	Infertile (Endometriosis)	NO	86.67	84.62	63.64	100.00
P30	19.70	12.70	0.00	Infertile (AMA)	YES	80.00	66.67	87.50	50.00
P31	150.00	2.60	3.00	Infertile (AMA)	YES	83.33	60.00	0.00	NA
P32	6.96	43.24	5.00	Infertile (AMA)	YES	100.00	0.00	NA	NA
P33	71.82	59.36	0.00	Infertile (AMA)	YES	91.67	54.55	100.00	50.00
P34	9.82	53.85	5.50	Infertile (AMA)	YES	42.86	66.67	50.00	NA
P35	18.11	26.38	1.00	Infertile (AMA)	YES	70.00	28.57	0.00	0.00
P36	19.87	7.20	2.00	Fertile	NO	80.00	83.33	60.00	100.00
P37	28.63	43.18	1.00	Infertile (AMA)	YES	57.14	75.00	100.00	NA
P38	121.90	13.00	NA	Infertile (AMA)	YES	50.00	66.67	0.00	0.00
P39	11.86	38.30	40.00	Infertile (AMA)	YES	80.00	33.33	50.00	NA
P40	15.43	50.47	6.00	Fertile	NO	100.00	100.00	0.00	100.00

NA = not applicable. AMA = advanced maternal age.

## Data Availability

Data is contained within the article or Supplementary Materials.

## Supplementary Materials

The following supporting information can be downloaded at: https://www.mdpi.com/article/10.3390/ijms26136314/s1.

## Abbreviations

The following abbreviations are used in this manuscript:

ART	Assisted reproductive technologies
SDF	Sperm DNA fragmentation
SD	Standard deviation
ROC	Receiver operating characteristic
AUC	Area under the curve
WHO	World Health Organization
SCSA	Sperm chromatin structure assay
SCD	Sperm chromatin dispersion
TUNEL	Terminal deoxynucleotidyl nick-end labeling
CI	Confidence interval
PPV	Positive predictive value
NPV	Negative predictive value
AMA	Advanced maternal age
NA	Non applicable
PI	Propidium iodide
PE	Phycoerythrin
FITC	Fluorescein Isothiocyanate
