# Supplementary material for: The Utility of Sperm DNA Fragmentation as a Diagnostic Tool for Male Infertility and Its Predictive Value for Assisted Reproductive Technology Outcomes
Source: Int J Mol Sci. 2025 Jun 30;26(13):6314. doi: 10.3390/ijms26136314 (PMC12250217; doi:10.3390/ijms26136314)
Supplement: Supplementary file 1 [file ijms-26-06314-s001.zip › ijms-3680491-supplementary.pdf]

Supplementary Materials

Supplementary Figure S1. Flow cytometry gating strategy. (a) Sperm-cell selection and debris exclusion; (b) Single sperm cell selection; (c) Apoptotic bodies exclusion through propidium iodide (PI)+ cell selection; (d) SDF+ cell selection based on Fluorescein Isothiocyanate (FITC) labelling; (e) Negative control threshold setting; (f) Positive control threshold setting. SSC = Side scatter; FSC-A = Forward scatter area; FSC-H = Forward scatter height; PE = Phycoerythrin

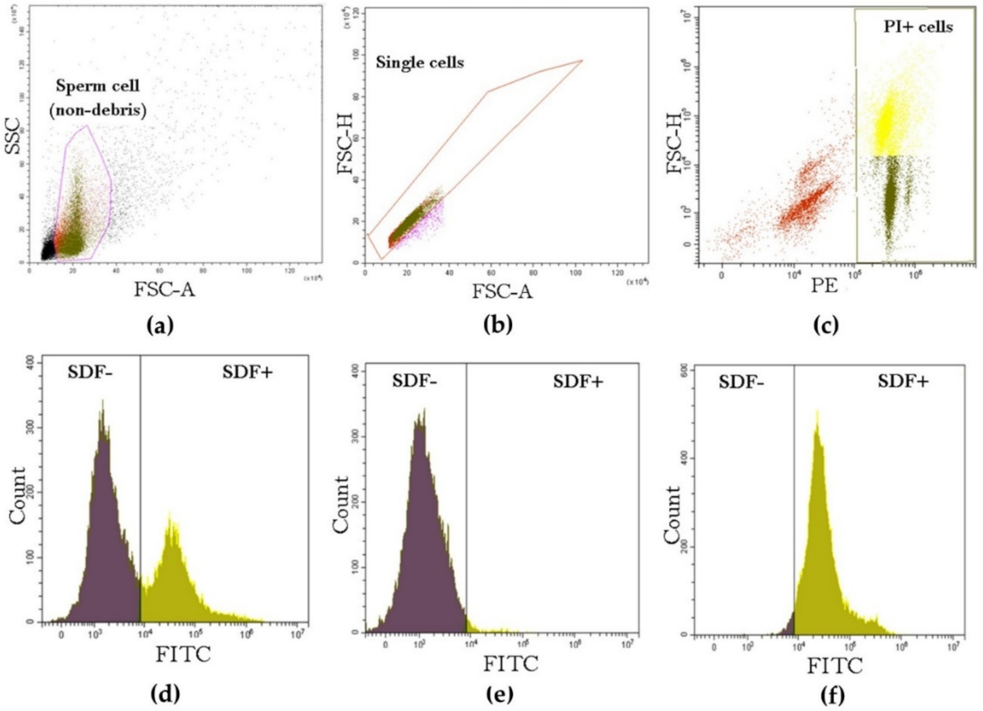

Supplementary Table S1. SDF results in fertile donors along with their corresponding thresh-old-based classification (high/low). The 95% confidence interval (CI) limits, and the cut-off value derived for binary classification are specified.

CONTROL SUBJECT ID	Acquired sperm	SDF- sperm	SDF+ sperm	SDF (%)	Threshold-Based Classification
**D1**	10362	8116	2246	21.68	Low
**D2**	10022	7548	2474	24.69	Low
**D3**	19996	17632	2364	11.82	Low
**D4**	10018	8271	1747	17.44	Low
**D5**	11161	8280	2881	25.81	Low
**D6**	10603	7856	2747	25.91	Low
**D7**	10411	7761	2650	25.45	Low
**D8**	9954	7141	2813	28.26	High
**D9**	9872	8054	1818	18.42	Low
**D10**	12474	8624	3850	30.86	High
**D11**	4465	2822	1643	36.8	High
**D12**	10668	9887	781	7.32	Low
**D13**	10929	8893	2036	18.63	Low
**D14**	11912	9479	2433	20.42	Low
**D15**	10281	7417	2864	27.86	High
**D16**	12985	9385	3600	27.72	High
**D17**	9952	6352	3600	36.17	High
**D18**	10105	8330	1775	17.57	Low
**D19**	10012	8590	1422	14.20	Low
**D20**	9981	9303	678	6.79	Low
**Mean**	NA	NA	NA	22.19	NA
**95% CI**	NA	NA	NA	18.27-26.11	NA
**Cut-off value**	NA	NA	NA	26.00	NA

Supplementary Table S2. SDF results in infertile patients along with their corresponding threshold-based classifications (high/low).

INFERTILE SUBJECT ID	Acquired sperm	SDF- sperm	SDF+ sperm	SDF (%)	Threshold-Based Classification
P1	9576	7866	1710	17.86	Low
P2	10182	9069	1113	10.93	Low
P3	8782	4195	4587	52.23	High
P4	10203	6537	3666	35.93	High
P5	10381	8369	2012	19.38	Low
P6	11767	9302	2465	20.95	Low
P7	10283	6620	3663	35.62	High
P8	11497	8532	2965	25.79	Low
P9	9993	8210	1783	16.14	Low
P10	10495	6481	4014	38.25	High
P11	15897	13370	2527	15.90	Low
P12	10316	6904	3412	33.07	High
P13	12566	10660	1906	15.17	Low
P14	6601	4133	2468	37.39	High
P15	11488	7109	4379	38.12	High
P16	15281	11119	4162	27.24	High
P17	16292	10352	5940	36.46	High
P18	4334	1242	3092	71.34	High
P19	12995	8613	4382	33.72	High
P20	10180	9072	1108	10.88	Low
P21	1058	567	491	46.41	High
P22	11305	8403	2902	25.67	Low
P23	13199	6933	6266	47.47	High
P24	12660	7296	5364	42.37	High
P25	10000	5364	4636	46.36	High
P26	11172	4406	6766	60.56	High
P27	9808	6449	3359	34.25	High
P28	10953	6806	4147	37.86	High
P29	8953	5133	3820	42.67	High
P30	5703	4306	1397	24.50	Low
P31	10002	6898	3104	31.03	High
P32	11707	9262	2445	20.88	Low
P33	10000	8178	1822	18.22	Low
P34	9982	6668	3314	33.20	High
P35	9845	5503	4342	44.10	High
P36	11791	6836	4955	42.02	High
P37	16381	12277	4104	25.05	Low
P38	12236	6178	6058	49.51	High
P39	10652	8029	2623	24.62	Low
P40	9719	7626	2093	21.54	Low
